# Supplementary material for: PLASMA: Partial LeAst Squares for Multiomics Analysis
Source: Cancers (Basel). 2025 Jan 17;17(2):287. doi: 10.3390/cancers17020287 (PMC11763701; doi:10.3390/cancers17020287)
Supplement: Supplementary file 1 [file cancers-17-00287-s001.zip › cancers-3383907-supplementary.pdf]

Supplemental Materials for ‘PLASMA: Partial LeAst Squares for Multiomics Analysis’

Kyoko Yamaguchi [1], Salma Abdelbaky [1], Lianbo Yu [2], Christopher C. Oakes [1], Lynne V. Abruzzo [3] and Kevin R. Coombes [4,*]

[1]Division of Hematology, Department of Internal Medicine, Ohio State University, Columbus, OH 43210, USA
[2]Department of Biomedical Informatics, Ohio State University, Columbus, OH 43210, USA
[3]Department of Pathology and Laboratory Medicine, Medical University of South Carolina, Charleston, SC 29425, USA
[4]Department of Biostatistics, Data Science, and Epidemiology, School of Public Health, Georgia Cancer Center at Augusta University, Augusta, GA 30912, USA

* Correspondence: kcoombes@augusta.edu

Supplemental Results

Supplemental Figure S1

Figure S1 shows that there is no significant difference in survival between the training and test sets formed by splitting the STAD TCGA data.

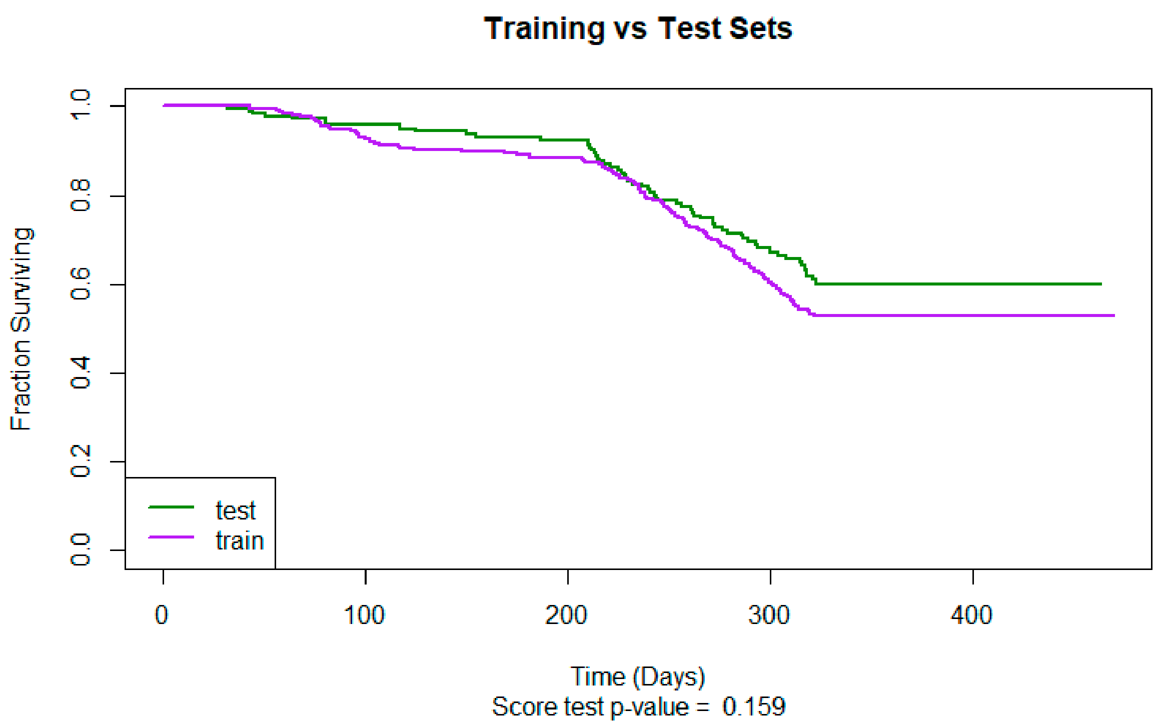

Supplemental Figure S1. Kaplan–Meier plot of overall survival for the training and test sets.

Supplemental Figure S2

Figure S2 presents the performance of individual omics models on the STAD test data.

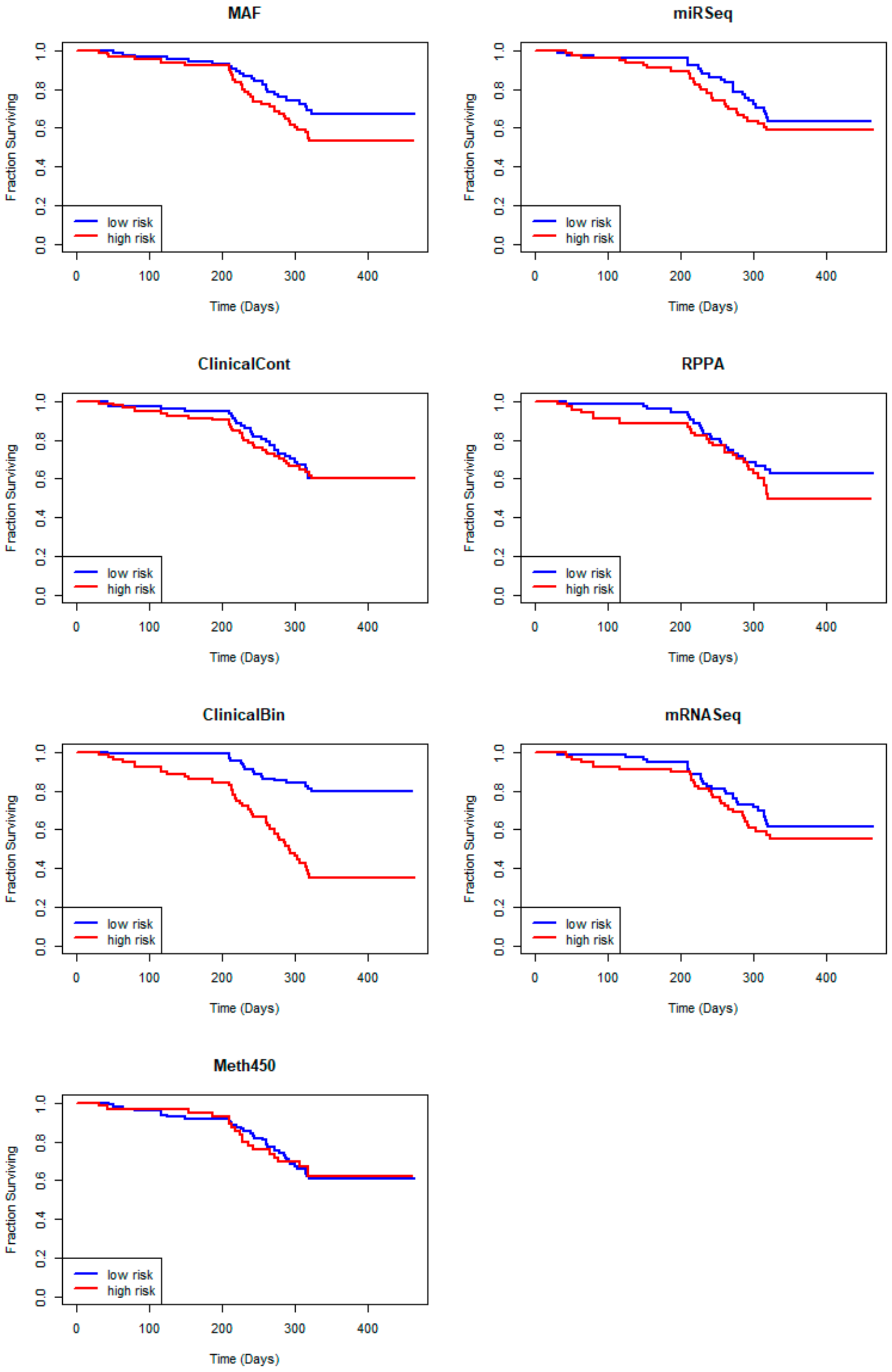

Supplemental Figure S2. Kaplan–Meier plots of overall survival on the STAD test set from separate PLS Cox omics models.

Supplemental Figure S3

Figure S3 is a heatmap equivalent to **Figure 7** in the main text, using correlation as the similarity measure to drive clustering. The vertical dendrogram makes it clear that the biological components associated with the "final" joint model are most strongly correlated with those associated to "all genes" used across individual components. The color scheme also highlights the fact that biological processes in the center part of the plot tend to be strongly associated with all components used in producing the final prognostic model.

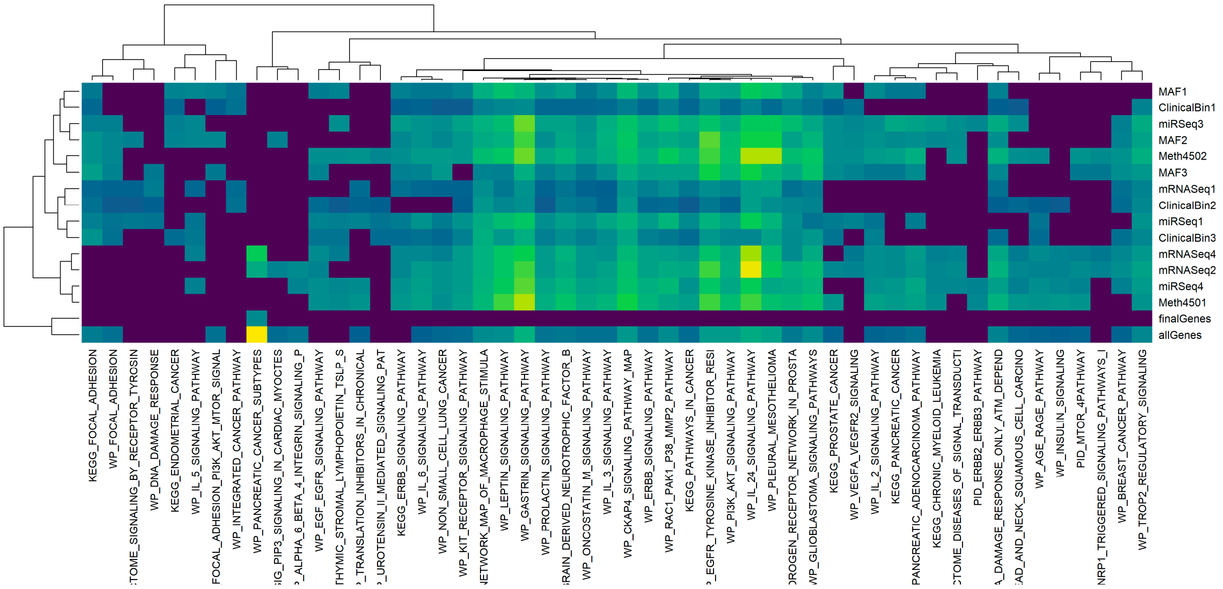

Supplemental Figure S3. Components clustered based on biological process annotation p-values.

Supplemental Figure S4

Figures S4–S7 are bubble plots similar to **Figure 7** in the main text, showing the association of the components with different kinds of gene sets.

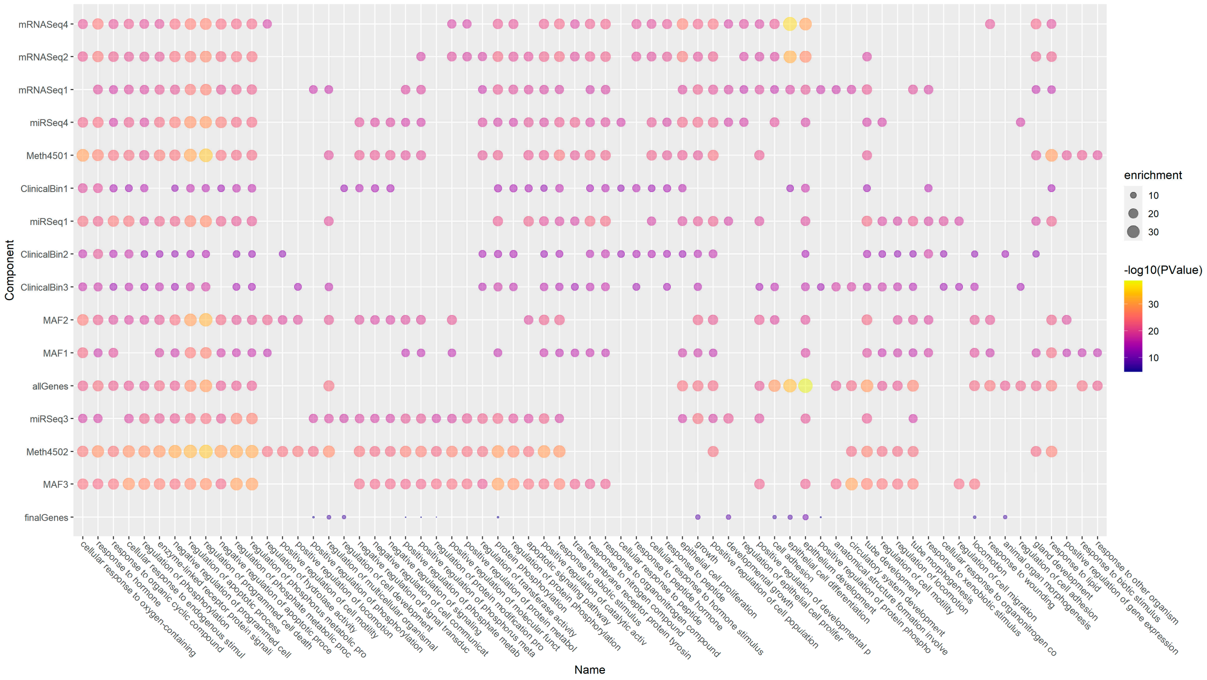

Supplemental Figure S4. Components clustered based on biological process annotation p-values.

Supplemental Figure S5

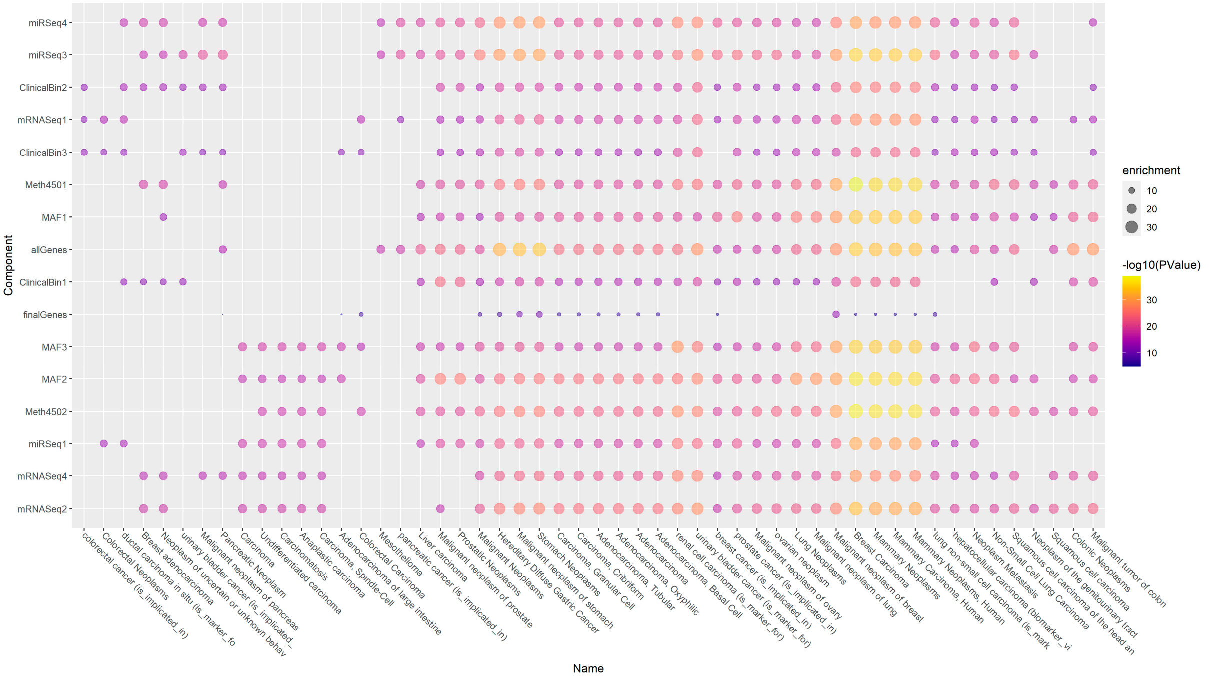

Supplemental Figure S5. Components clustered based on disease annotation p-values.

Supplemental Figure S6

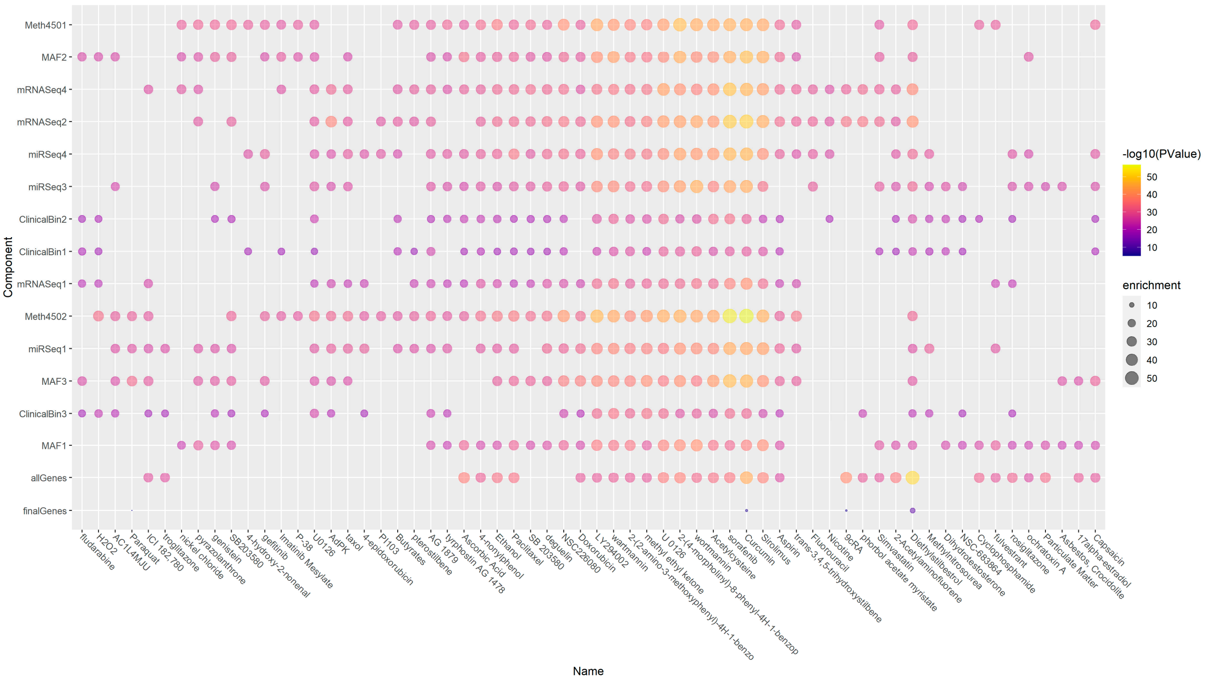

Supplemental Figure S6. Components clustered based on drug annotation p-values.

Supplemental Figure S7

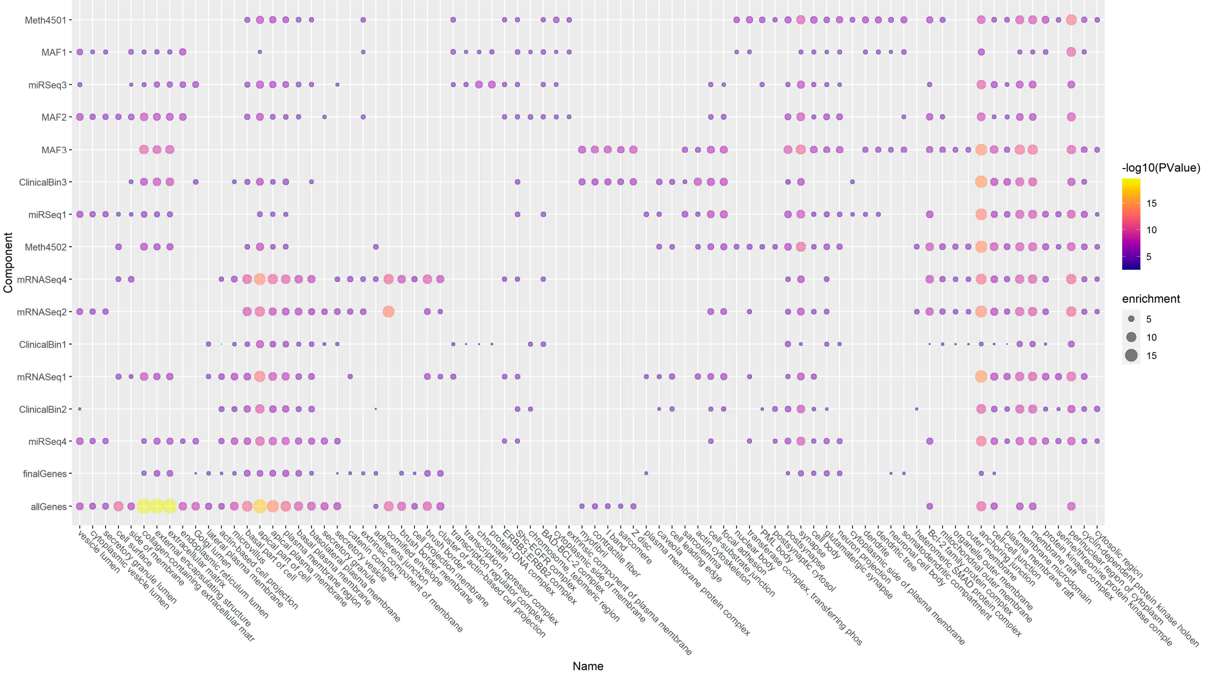

Supplemental Figure S7. Components clustered based on cellular component annotation p-values.

MOFA and Survival

Here, we run Multiomics Factor Analysis (MOFA) to illustrate the differences between a supervised analysis (as provided by our `plasma` package) and a mainly unsupervised analysis of multiomics data to predict time-to-event outcomes. By the latter, we specifically mean

1. Running MOFA to detect latent factors across multiple omics data sets.
2. Testing the latent factors on new data to see if they are at all associated with the time-to-event outcome that we want to be able to predict.

We load the same version of the data, already split into training and test sets, that we used for our `plasma` analysis in the main text. Then we create the initial MOFA object.

Supplemental Figure S8

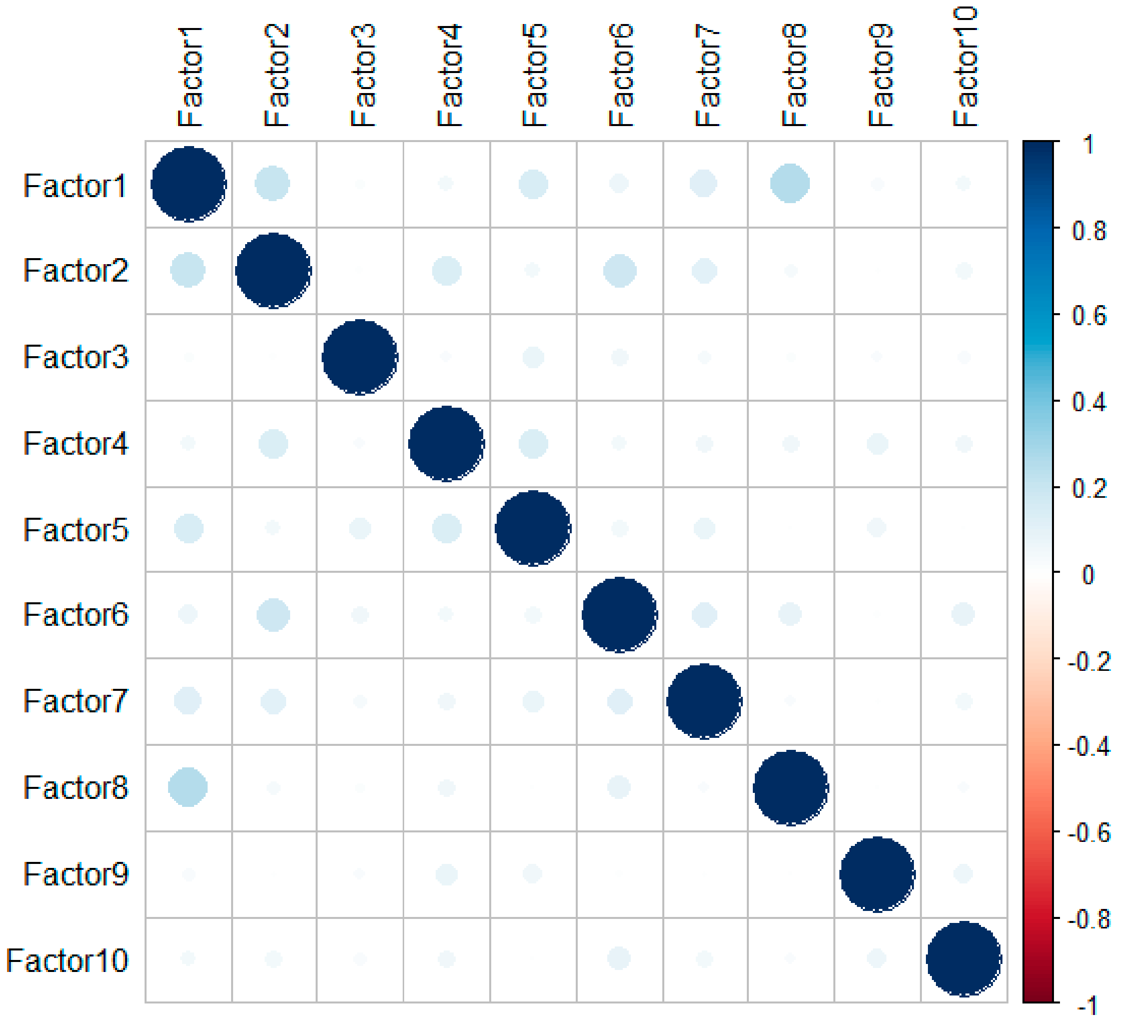

Supplemental Figure S8. Correlation between MOFA latent factors.

Supplemental Figure S9

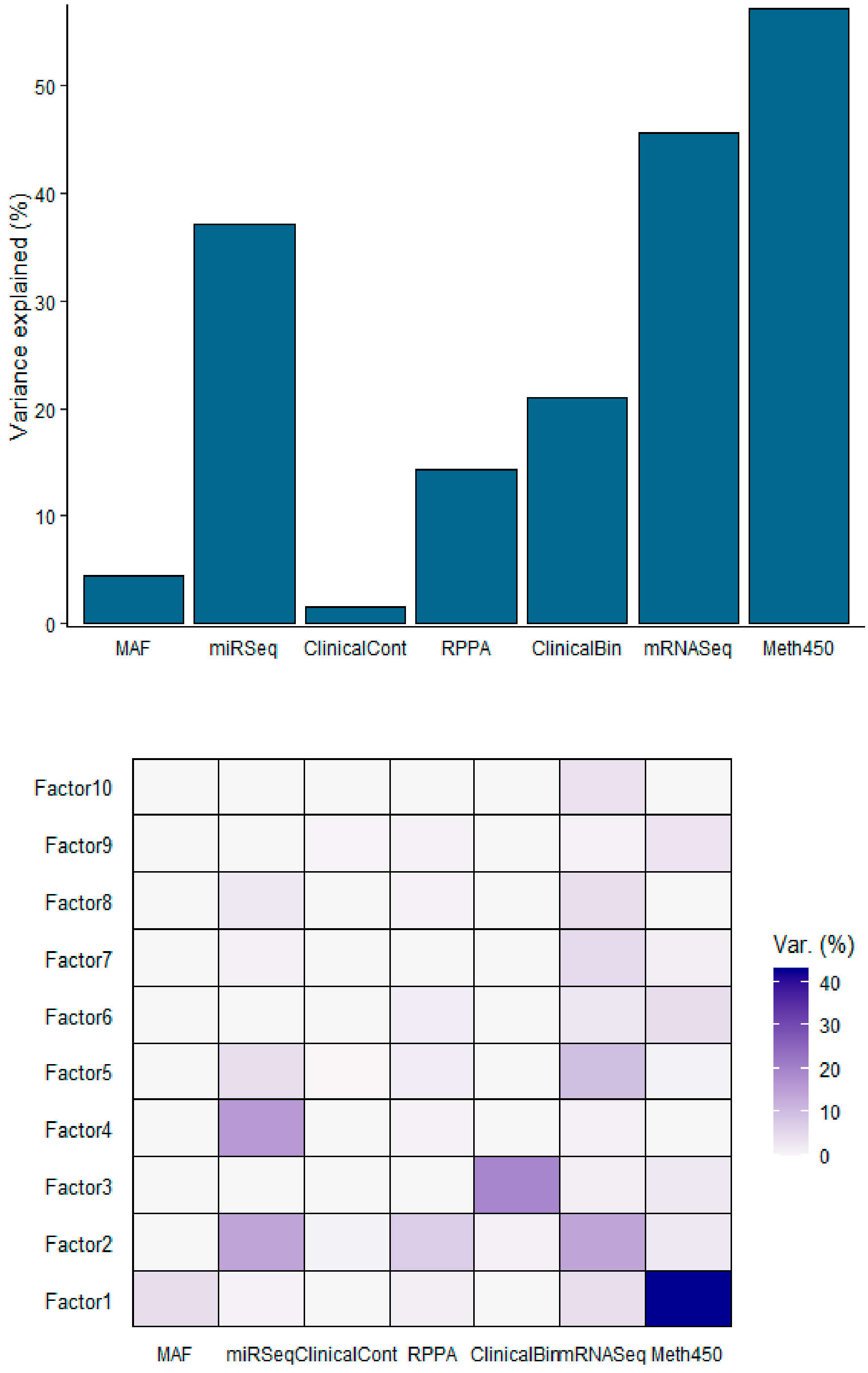

Supplemental Figure S9. Amount of variance explained by latent factors in omics data sets.

Training: Relationship to Overall Survival

We want to see whether any of the latent factors are associated with overall survival. We start by fitting separate univariate models for each of the latent factors. The results of these univariate models suggest that only Factors 2, 10, and 4 are even marginally useful for prediction (**Table S1**).

Table S1. Details of the univariate Cox Proportional Hazards models for overall survival (OS) using MOFA factors as predictors (Coef = log hazard ratio, HR = hazard ratio, score = log-rank test statistic).

	coef	HR	score	pvalue
Factor1	0.0460390	1.0471153	0.9122469	0.3395189
Factor2	0.0969772	1.1018352	2.5846875	0.1079017
Factor3	0.0217074	1.0219448	0.0750634	0.7841023
Factor4	-0.1168017	0.8897616	1.4352500	0.2309095
Factor5	0.0409005	1.0417485	0.2412707	0.6232898
Factor6	0.0089701	1.0090104	0.0086321	0.9259759
Factor7	0.0862323	1.0900596	0.8023007	0.3704064
Factor8	-0.0190968	0.9810844	0.0446093	0.8327241
Factor9	0.0647337	1.0668749	0.4330933	0.5104750
Factor10	0.0948066	1.0994462	1.5297208	0.2161543

Next, we construct a multivariate model combining all of the factors as possible predictors of overall survival. The p-values associated with individual factors in the multivariate model again suggest that, at best, only Factors 2 and 4 might be useful (**Table S2**).

Table S2. Details of the multivariate Cox Proportional Hazards model for overall survival (OS) using all MOFA factors as predictors (coef = log hazard ratio, exp(coef = hazard ratio. se = standard error).

	coef	exp(coef)	se(coef)	z	Pr(>|z|)
Factor1	0.0177368	1.0178950	0.0548793	0.3231966	0.7465463
Factor2	0.1140809	1.1208428	0.0669179	1.7047875	0.0882341
Factor3	0.0433160	1.0442678	0.0801522	0.5404217	0.5889062
Factor4	-0.1488849	0.8616683	0.0992794	-1.4996561	0.1337035
Factor5	0.0479219	1.0490888	0.0842644	0.5687093	0.5695534
Factor6	-0.0391104	0.9616445	0.1038272	-0.3766879	0.7064055
Factor7	0.0727848	1.0754990	0.1050473	0.6928765	0.4883870
Factor8	-0.0118319	0.9882379	0.0915264	-0.1292726	0.8971419
Factor9	0.0749702	1.0778521	0.0973015	0.7704946	0.4410066
Factor10	0.1065461	1.1124292	0.0755382	1.4104933	0.1583941

Finally, we use the Akaike Information Criterion (AIC) to formally select factors to retain in the final model. This process confirms our earlier suspicions that only Factors 2 and 4 make useful contributions to the model (**Table S3**). Starting with the predicted risk scores from the AIC model, we cut the scores at 0 to create "low" and "high" risk cohorts. The resulting Kaplan-Meier plot is shown in **Figure S10**. The model has a not-quite significant separation between the two risk groups (p = 0.06) in the training set.

Table S3. Details of the multivariate Cox Proportional Hazards model for overall survival (OS) with variables selected using stepwise regression with AIC.

	coef	exp(coef)	se(coef)	z	Pr(>|z|)
Factor2	0.1119490	1.118456	0.0621418	1.801508	0.0716228
Factor4	-0.1402108	0.869175	0.0962488	-1.456753	0.1451846

Supplemental Figure S10

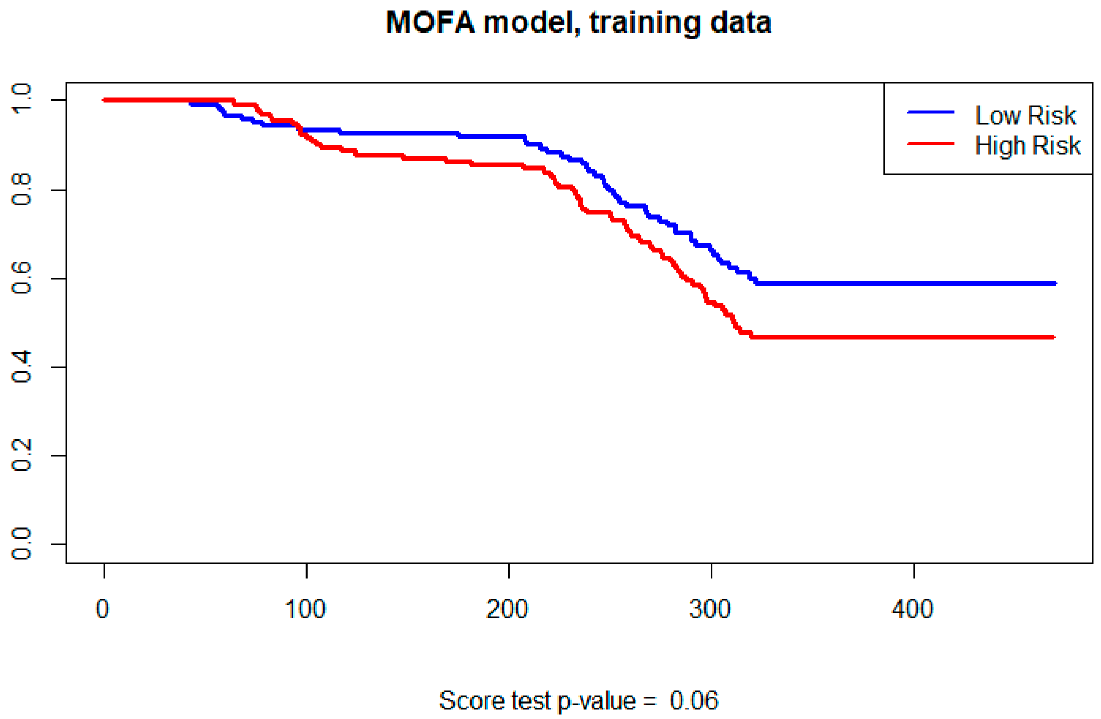

Supplemental Figure S10. Kaplan–Meier plot based on risk predicted by MOFA latent factors.

Approximating the MOFA Computations

The hard part is to reverse-engineer the MOFA model so we can use its decompositions to make predictions on the test data. The statistical model underlying MOFA is that the matrix Z of latent factors should simultaneously satisfy the equations $X_i = W_i Z$ for each omics data matrix X_i and for appropriate weight matrices W_i. In principle, this means that we should (at least approximately) be able to recompute Z from the original data matrices and the known weight matrices.

First, we illustrate two ways to recover the latent factor matrix Z.

```
Z0 <- get_factors(mofaSTADedit)[[1]]
Z <- mofaSTADedit@expectations$Z[[1]]
identical(Z0, Z) # TRUE

## [1] TRUE
```

Next, we illustrate two ways to recover the weight matrices, W_i.

```
weightmats <- get_weights(mofaSTADedit)
identical(weightmats, mofaSTADedit@expectations$W) # TRUE

## [1] TRUE

Woo <- do.call(rbind, weightmats)
```

The last line in the previous code bundles the weight matrices together into a single matrix W.

Because MOFA internally centers the data in continuous input matrices, we have to do the same to the input training data.

So, we bundle the (centered) input data matrices into a single matrix, X. We also replace all of the missing data (which arises as complete rows when a patient has not been included in an assay) with zeros. Then we solve for Z by using a QR-decomposition of the full weight matrix.

Supplemental Figure S11

In **Figure S11**, we show that the approximation that we have constructed for Z still yields a model that separates the training data into low- and high-risk patients. Because this only approximates the actual MOFA latent factors, this model is even less significant (p=0.11) than the previous one.

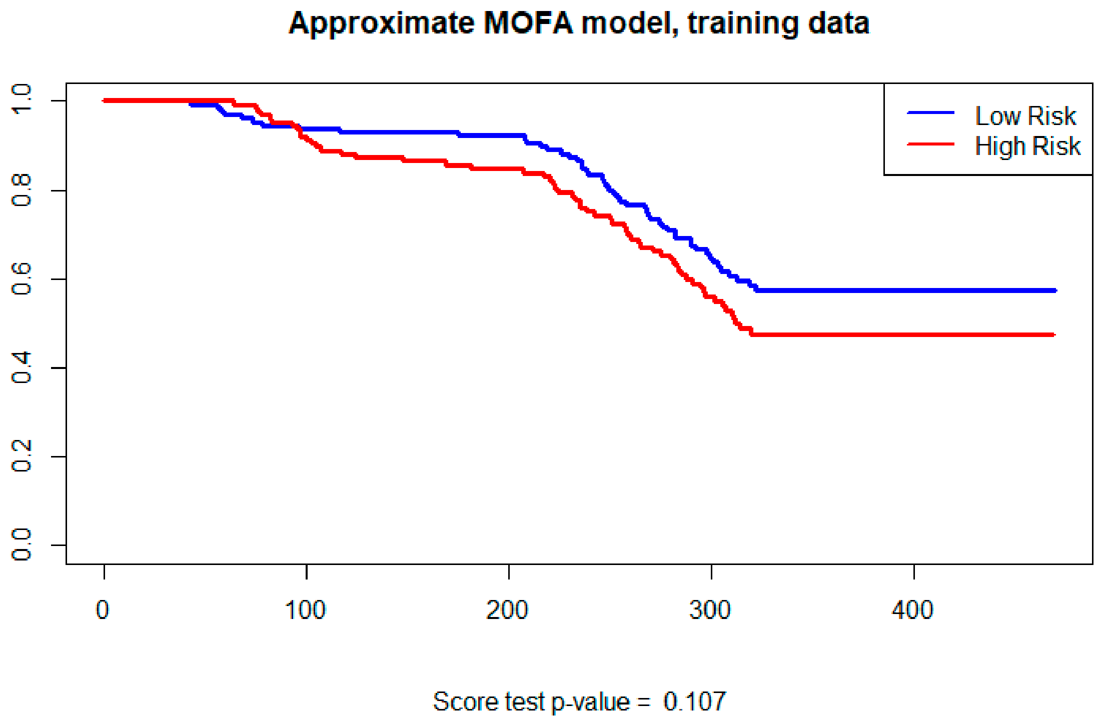

Supplemental Figure S11. Kaplan–Meier plot of approximate MOFA model on the training data.

Test Data

Now we can use the same weight matrices that we learned on the training data to project the independent test data into the latent factor space.

Then we split the predicted scores from the AIC model applied to the test data at the same cutoff (i.e., zero) that we used for training.

Supplemental Figure S12

Surprisingly (since the model was not significant on the training set, it does seem to do a passable job on the independent test data, with p = 0.016 (**Figure S12**)). This result is somewhat disconcerting. Any sort of learned model isn't supposed to perform *better* on the test set than it does on the training set. We suspect that we were just fortunate.

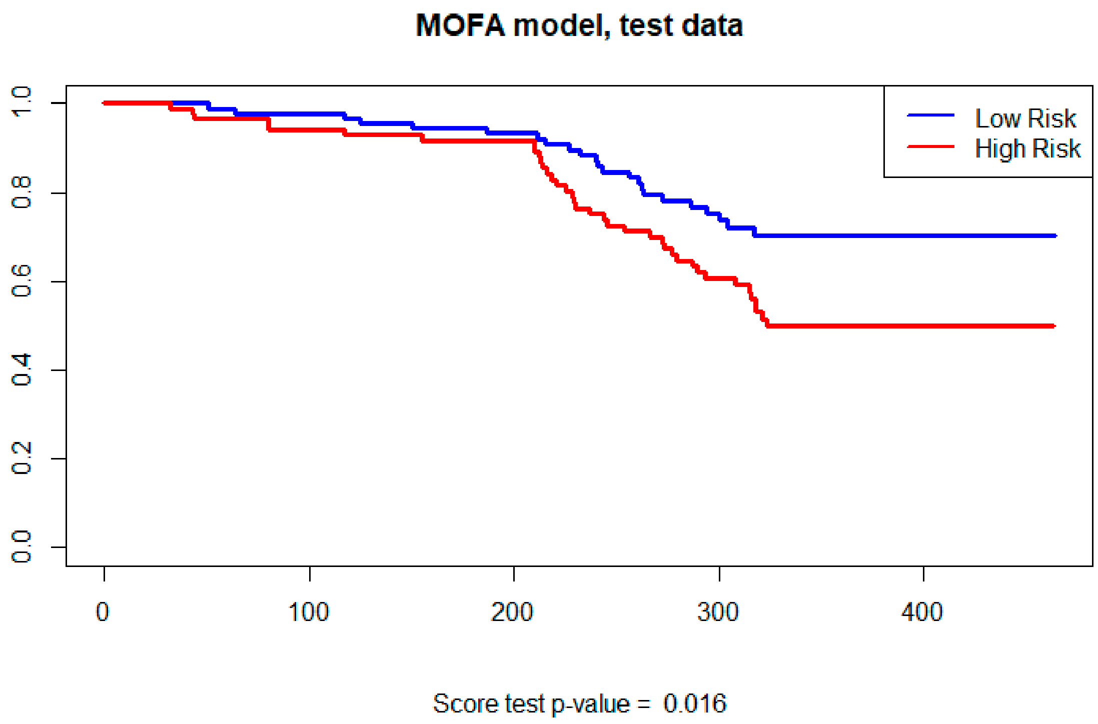

Supplemental Figure S12. Kaplan–Meier plot of MOFA model on test data.

Independent Validation with ESCA Adenocarcinoma Data

Finally, we apply the same model that we learned from the STAD training data to the adenocarcinoma subset of the esophageal cancer (ESCA) data from TCGA.

Supplemental Figure S13

Figure S13 shows that the model is completely unable to predict prognosis in the ESCA adenocarcinoma data set (p = 0.237)

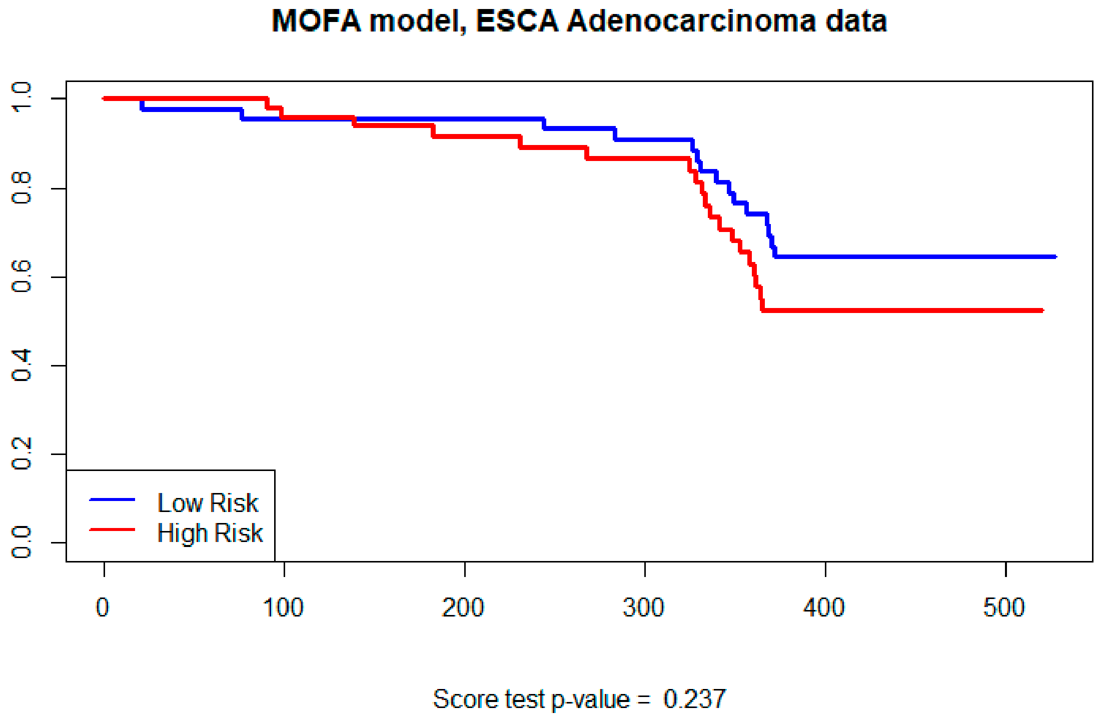

Supplemental Figure S13. Kaplan–Meier plot of MOA model on the ESCA adenocarcinoma validation data.
